# Independent evolution of macrophage-tropism and increased charge between HIV-1 R5 envelopes present in brain and immune tissue

**DOI:** 10.1186/1742-4690-9-20

**Published:** 2012-03-15

**Authors:** Maria Paz Gonzalez-Perez, Olivia O'Connell, Rongheng Lin, W Matthew Sullivan, Jeanne Bell, Peter Simmonds, Paul R Clapham

**Affiliations:** 1 Program in Molecular Medicine and Department of Microbiology and Physiological Systems, University of Massachusetts Medical School, Biotech 2, 373 Plantation Street, Worcester, Massachusetts 01605-2377; 2 School of Public Health and Health Sciences, University of Massachusetts, 411 Arnold House, 715 North Pleasant Street, Amherst, MA 01003-9304; 3 The MRC HIV brain and tissue bank in Edinburgh, Department of Pathology (Neuropathology), University of Edinburgh, Wilkie Building, Teviot Place, Edinburgh, UK EH8 9AG; 4 Centre for Infectious Diseases, Summerhall, Edinburgh EH9 1QH, UK

**Keywords:** HIV, Envelope, Macrophage-tropism, CD4, CCR5, Neurotropism, Immune tissue, Brain, Entry

## Abstract

**Background:**

Transmitted HIV-1 clade B or C R5 viruses have been reported to infect macrophages inefficiently, while other studies have described R5 viruses in late disease with either an enhanced macrophage-tropism or carrying envelopes with an increased positive charge and fitness. In contrast, our previous data suggested that viruses carrying non-macrophage-tropic R5 envelopes were still predominant in immune tissue of AIDS patients. To further investigate the tropism and charge of HIV-1 viruses in late disease, we evaluated the properties of HIV-1 envelopes amplified from immune and brain tissues of AIDS patients with neurological complications.

**Results:**

Almost all envelopes amplified were R5. There was clear compartmentalization of envelope sequences for four of the five subjects. However, strong compartmentalization of macrophage-tropism in brain was observed even when brain and immune tissue envelope sequences were not segregated. R5 envelopes from immune tissue of four subjects carried a higher positive charge compared to brain envelopes. We also confirm a significant correlation between macrophage tropism and sensitivity to soluble CD4, a weak association with sensitivity to the CD4 binding site antibody, b12, but no clear relationship with maraviroc sensitivity.

**Conclusions:**

Our study shows that non-macrophage-tropic R5 envelopes carrying gp120s with an increased positive charge were predominant in immune tissue in late disease. However, highly macrophage-tropic variants with lower charged gp120s were nearly universal in the brain. These results are consistent with HIV-1 R5 envelopes evolving gp120s with an increased positive charge in immune tissue or sites outside the brain that likely reflect an adaptation for increased replication or fitness for CD4+ T-cells. Our data are consistent with the presence of powerful pressures in brain and in immune tissues selecting for R5 envelopes with very different properties; high macrophage-tropism, sCD4 sensitivity and low positive charge in brain and non-macrophage-tropism, sCD4 resistance and high positive charge in immune tissue.

## Background

Human immunodeficiency virus type 1 requires interactions with CD4 and either CCR5 or CXCR4 coreceptors to trigger fusion of viral and cellular membranes and entry into cells. CCR5-using (R5) viruses are mainly transmitted and individuals homozygous for a defective CCR5 gene (Δ32 CCR5) are substantially protected from infection [1]. Recent studies of clade B and clade C transmission events have shown that the transmitted R5 viruses are unable to efficiently infect macrophages, whether transmission is sexual [2,3] or via mother-to-child [4]. In late disease, CXCR4-using (X4) variants can be isolated from up to 50% of AIDS patients and are associated with a more rapid loss of CD4^+ ^T-cells and faster disease progression [5-8]. However, whether R5 viruses evolve distinct properties that impact on pathogenesis is poorly understood. R5 viruses with an enhanced macrophage-tropism were isolated from adult [9,10] and pediatric [11] AIDS patients who did not develop CXCR4-using variants. However, our previous data indicated that viruses carrying non-macrophage-tropic R5 envelopes were present in immune tissue (lymph nodes) in late disease, even in subjects with neurological complications who carried highly macrophage-tropic variants in brain tissue [12,13]. Finally, other groups reported that envelopes with an increased positive charge, fitness and reduced sensitivity to CCR5 inhibitors [14-17] evolve in late disease. Whether increased macrophage-tropism and envelope charge are separate or related properties has not been extensively researched, although in a preliminary study, we did not detect a correlation [18]. The current study was designed to investigate the relationship between macrophage-tropism and gp120 charge for HIV-1 R5 envelopes present in immune and brain tissue of AIDS patients with neurological issues.

Untreated HIV-1+individuals frequently suffer from HIV associated neurocognitive disorders, which are characterized by sensory neuropathy, sensory myelopathy and eventually dementia. The most severe dementias occur in about 30% of AIDS patients. The mechanisms that cause dementia are unclear but likely involve disruption of normal neurological functions by toxic factors that are upregulated either as a direct result of HIV replication or indirectly as a consequence of inflammatory processes [19-21]. Even in the era of highly effective anti-retroviral therapies, milder neurocognitive impairments persist [22,23], while the more severe neurocognitive disorders are still apparent in subjects who fail therapy [24].

The brain is colonized early after infection [25]. However, proviral DNA is difficult to detect in brain tissue during the asymptomatic phase [26-29]. Mechanisms of entry into the brain are unclear, although the virus must penetrate the blood brain barrier (BBB) or enter via the choroid plexus and cerebral spinal fluid (CSF). A 'trojan horse' mechanism of entry is favored whereby infected monocytes carry the virus through the BBB [30]. In support of this, HIV env sequences in deep white matter were reported to be more closely related to env sequences in blood monocytes than sequences from other tissues [31,32], although only a single individual was studied. *In situ *hybridization and immunohistochemistry have detected HIV infection and accumulation in cells surrounding blood capillaries including CD14^+ ^cells [33-35]. These CD4^+ ^CCR5^+ ^perivascular macrophages are a major reservoir of HIV-1 in the brain [19,36-38]. A hallmark of HIV-associated neuropathology is the presence of multinucleated giant cells (MNGCs) in brain tissue [19,39]. MNGCs express CD68 and are believed to result from HIV induced fusion of infected and uninfected perivascular macrophages [40]. Resident microglia (monocyte/macrophage lineage) are also infected in humans [36,37,41] and support HIV replication when cultured *in vitro *[19]. The number of activated monocytes in blood was reported to correlate with AIDS dementia [42], and it is likely that the brain is reseeded by infected, activated monocytes that enter the brain in late disease [32].

Many studies have shown that HIV-1 sequences are frequently compartmentalized in the brain and are distinct from sequences from blood or immune tissue [43-49]. A relatively small number of highly macrophage-tropic R5 virus isolates made from brain tissue have been described [50,51]. Fewer studies have compared the phenotypes of envelopes derived by PCR without culture from these compartments. As discussed above, we reported that envelopes amplified from brain tissue were predominantly highly tropic for macrophages while those from other sites including blood, plasma, lymph node and spleen were predominantly non-macrophage-tropic despite uniformly using CCR5 and not CXCR4 [12,52]. The only other study to investigate the tropism of PCR amplified envelopes also described a wide variation in macrophage-tropism of R5 envelopes but failed to demonstrate a clear association of mac-tropism with brain envelopes compared to those from lymphoid tissue [53]. Furthermore, a potential role for CXCR4-using viruses in the brain has also been proposed [50,51,54,55]. It should also be emphasized that these earlier studies investigating the phenotypes of envelopes amplified from brain tissue studied relatively few envelopes from only a few individuals [12,13,53].

Here, we endeavored to evaluate the phenotypes of a large number of envelopes amplified by PCR from the brain and the immune tissue. We present a detailed investigation of 183 envelope clones amplified from brain, spleen and lymph node tissue of five subjects including four new subjects who died with HIV associated dementia. We show that HIV-1 R5 envelopes carrying gp120s with an increased positive charge were present in immune tissue and had evolved independently from highly macrophage-tropic variants with low charge gp120s in the brain. We also show that independent evolutionary pathways to macrophage-tropism are present in immune tissue. In summary, our data are consistent with the presence of powerful pressures in brain and in immune tissue selecting for (or against) R5 envelopes that are highly macrophage-tropic or that carry an increased gp120 charge respectively. Our study provides important new insights into the evolution of HIV-1 R5 envelopes with very distinct properties at different sites in the body and driven by powerful tissue specific evolutionary pressures.

## Results

### Compartmentalization of HIV-1 envelope sequences from brain, lymph node and spleen tissue

**Figure 1:**
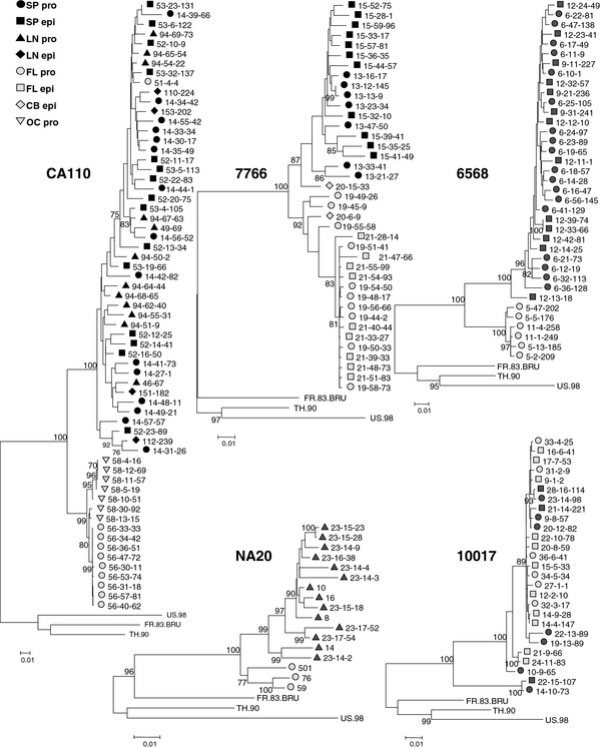
**Phylogenetic analysis (MEGA 5) of HIV-1 envelope nucleotide sequences amplified from brain and immune tissue**. Tight compartmentalization of brain and immune tissue envelope sequences was observed for subjects CA110, 6568, 7766 and NA20. Envelope compartmentalization was less clear for subject 10017. Numbers at branch points represent bootstrap values.

We first investigated the compartmentalization of envelope sequences (n = 183) amplified from proviral or episomal DNA of five HIV-1+ subjects with neurological conditions (Table 1). Circular episomal DNA has been reported to be labile in T-cells and associated with ongoing replication [56], although this is controversial [57]. A further report suggested that circular DNA forms were more stable in macrophages [58]. Envelope gp160 sequences were amplified from DNA extracted from frontal lobe tissue for all subjects, from the cerebellum for subject 7766 and occipital lobe for CA110. Envelopes were also amplified from the spleen of four subjects and from the lymph node of two including the fifth subject (NA20). Phylogenetic trees were produced using maximum likelihood methods via MEGA version 5. All envelopes amplified clustered with standard clade B envelopes and not with envelopes from other clades (data not shown). Trees presented on Figure 1 were rooted with three clade B envelope sequences. Envelope sequences for each subject segregated separately, were distinct from the three clade B reference sequences and did not segregate with any on the HIV sequence database http://www.hiv.lanl.gov/content/sequence/HIV/mainpage.html. For four subjects, brain envelopes were distinct from those from spleen (7766, CA110 and 6568) or lymph node (CA110 and NA20). Envelopes from the fifth subject (10017) were more interspersed. Envelopes amplified from circular DNA forms did not segregate separately from proviral DNA, regardless of tissue origin.

**Table 1:** Characteristics of HIV-1+ subjects studied

**Patient No**.	Collection Year	Brain Bank	Disease status	CD4 count	Viral Load*	Therapy history
7766	1999	NNTC	AIDS, HAD, HIVE	43	1,843	3TC, ABC, EFV, D4T, DDI, IDV, NFV, ZDV
6568	2001	NNTC	AIDS, HAD, HIVE	77	> 750,000	3TC, ABC, D4T, DDC, DDI, EFV, IDV, NFV, NVP, ZDV
CA110	1999	UCSD	AIDS, HAD, HIVE	21	198,957	No use reported
10017	1999	Mt. Sinai	AIDS, HIVE, IVDU	7	389,120	3TC, D4T, SQV, ZDV
NA20	1993	Edinburgh	Hemophiliac**	4	--	No use reported

*Last available record prior to death

**Mild cognitive impairment, sparse giant cell encephalitis, cerebral toxoplasmosis. Status = hemophiliac

### Almost all envelopes amplified were R5

All envelopes were screened for functionality by testing whether env+ pseudovirions conferred infection of HeLa TZM-bl cells, which express recombinant CD4 and CCR5 along with endogenous CXCR4. 56.9% of envelopes conferred high levels of infectivity for HeLa TZM-bl cells regardless of whether they were amplified from frontal lobe, spleen or lymph node. In contrast, envelopes amplified from the cerebellum of subject 7766 were not functional in pseudovirion assays. Some of the non-functional envelope carried premature stop codons. However, the majority had open reading frames and their lack of function was unclear.

CXCR4-use was tested on HeLa HIJ cells, which express CD4 and CXCR4 but not CCR5. Only envelope 22-15-107 (from the spleen of subject 10017) conferred low levels of HIJ infection likely indicating CXCR4-use. Thus, nearly all envelopes were R5 even though all the subjects under investigation had AIDS. Furthermore, all envelopes except for 22-15-107 and 14-10-73 (non-functional) were designated as R5 using the Web based PSSM program http://indra.mullins.microbiol.washington.edu/webpssm/ and carried overall positive charges of +1-5 (total of R and K residues minus total of E and D residues). Envelopes 22-15-107 and 14-10-73 (non-functional) carried a higher V3 charge (+7) consistent with CXCR4-use. These conclusions are further supported by inhibition data on HeLa TZM-bl cells for maraviroc (see below).

### Macrophage-tropism of envelopes from brain, spleen and lymph node tissue

**Figure 2:**
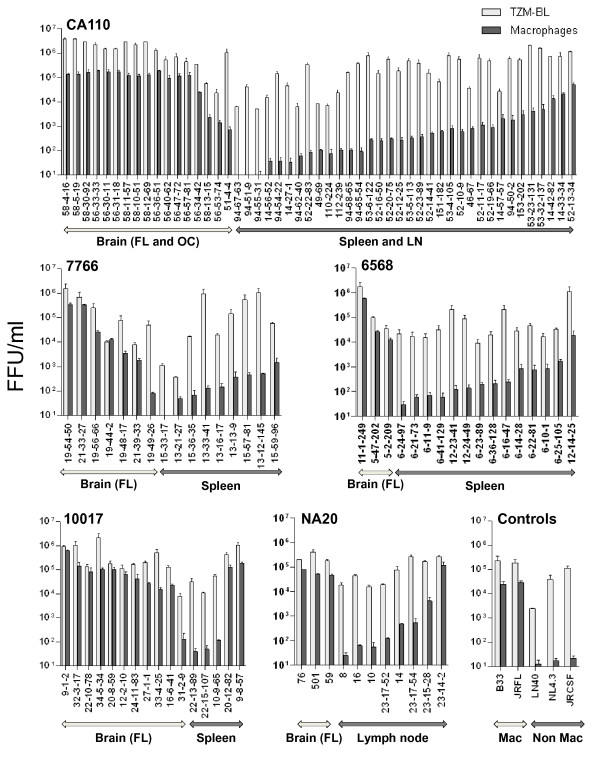
**Compartmentalized macrophage infectivity for brain and immune tissue envelopes**. Nearly all envelopes amplified from brain tissue efficiently infected primary macrophage cultures. In contrast, envelopes from immune tissue varied in the capacity to infect macrophages with infection ranging from background levels to highly efficient infection for a small minority. Infectivity is presented as FFU/ml and was derived from titers averaged from at least two assays using macrophage prepared from different donors.

We next investigated whether the ability of envelopes to infect primary macrophages was compartmentalized between the brain and immune tissue. We cannot be certain that envelopes amplified from brain tissue were derived from infected macrophages since astrocytes have also been reported to be infected [38,40,59-63]. Nevertheless, nearly all envelopes amplified from brain tissues conferred highly efficient infection of macrophages. In contrast, envelopes amplified from spleen or lymph node varied in macrophage-tropism from background levels up to modest levels of infection, and for a minority of envelopes, highly efficient infection (Figure 2). Thus, highly efficient macrophage infection was observed for subject 10017 spleen envelopes, 20-12-82 and 9-8-57, which clustered phylogenetically among a group of brain-derived envelopes (and along with three further spleen envelopes that were non-functional). However, for most subjects, envelopes from spleen or lymph node (that segregated separately from brain env sequences) showed a gradation in their capacity to infect macrophages ranging from background infection to modestly efficient levels, consistent with an independent evolutionary pathway to macrophage-tropism in immune tissue. In addition, the lymph node envelope 23-14-2 from NA20 conferred high levels of macrophage infection (similar to most brain envelopes), while clustering phylogenetically (Figure 1) with other immune tissue envelopes that conferred inefficient macrophage infection (Figure 2). This observation suggests that 23-14-2 evolved efficient macrophage infection independently from those in the brain. However, further analyses indicated that 23-14-2 is a recombinant between spleen and brain envelopes (data not shown).

A minority of brain-derived envelopes clustered closely with those from spleen; 20-15-33 (subject 7766) and 51-4-4 (subject CA110). Unfortunately, the 7766 envelope was not functional. However, 51-4-4 conferred a non-macrophage-tropic phenotype similar to spleen and lymph node envelopes, suggesting that it could have been derived from blood in brain tissue capillaries.

### Immune tissue envelopes carry a higher overall positive charge in V1-V2 and V1-V5 regions

**Figure 3:**
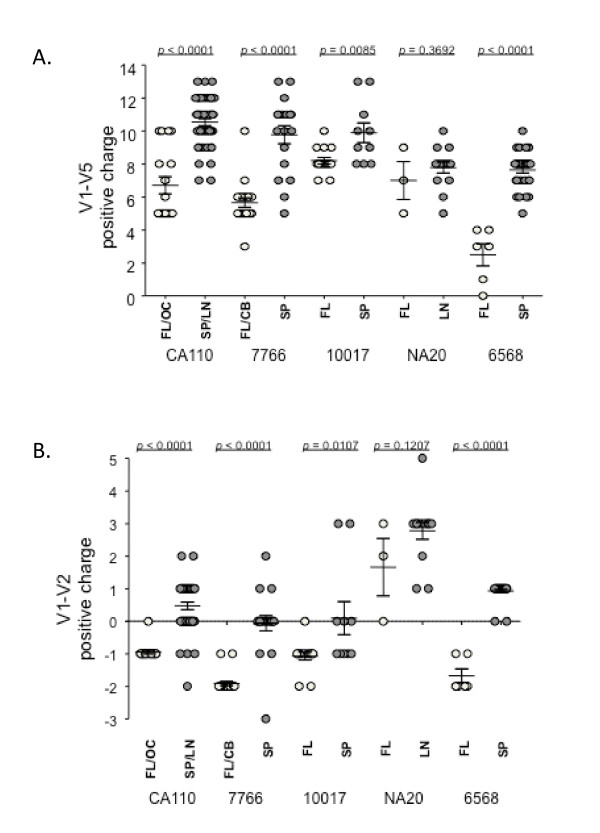
**Overall positive charge of HIV-1 V1-V2 and V1-V5 regions of envelopes amplified from brain and immune tissue**. Immune tissue envelopes from four of five subjects carry a significantly higher positive charge compared to corresponding envelopes from brain. A-B: Overall positive charge of V1-V5 (A) and V1-V2 (B) amino acid sequences. All amplified envelopes shown in Figure 1 were included in this analysis.

Previous studies reported two distinct and emerging phenotypes for primary R5 viruses isolated from blood in late disease. First, R5 isolates carrying a higher overall positive charge for the V1-V5 region were reported to carry increased fitness and decreased sensitivity to CCR5 ligands [14-17]. These properties were hypothesized to result in enhanced envelope interactions with CCR5 and increased replication in T-cells [14-17] including populations expressing lower CCR5 levels [64]. Second, several studies reported on the isolation of R5 isolates from blood of adult and pediatric AIDS patients that conferred an enhanced macrophage-tropism [9-11]. Here, we showed significant differences in the overall positive charge of V1-V5 and V1-V2 amino acids for 4 of the 5 subjects (Figure 3A, B). Brain-derived envelopes from these four subjects carried a lower overall charge compared to those from immune tissue (Table 2). For the fifth subject (NA20), there were insufficient brain envelopes to undertake a reasonable comparison. These observations show for the first time that these distinct envelope characteristics arise independently. R5 variants with increased gp120 charge appear to evolve in immune tissue while highly mac-tropic variants predominantly emerge in the brain and possibly other non-immune tissues where macrophages are the main targets for infection.

**Table 2:** Characteristics of envelopes amplified from brain and immune tissue

Subject	Tissue	N° of Amplicons	Average length (amino acids)			Average Overall Positive Charge			N° of CHO V1-V5*	Presence of N283 (%)	Presence of N386 (%)
						
			V1-V2	V1-V5	V3	V1-V2	V1-V5	V3			
CA110	FL	10	68.7	337.8	35	-0.9	5.3	3	22.7	100	100
	OC	7	69	338	35	-1	8.7	3	21	100	100
	SP	30	66.9	338.4	35	0.6	10.7	3.5	21.2	100	100
	LN	16	65.9	338	35	0.3	10.2	3.6	20.5	100	100
7766	FL	19	67.1	342.8	35	-2	5.5	1.8	18.8	100	100
	CB	2	68	344	35	-1.5	7.5	2	19	100	100
	SP	18	67.4	341.6	35	0	9.8	1.9	18.3	100	100
6568	FL	6	65	335	34	-1.7	2.5	2.8	17.7	0	0
	SP	31	68.1	338.4	35	0.9	7.6	1.1	15.9	0	100
10017	FL	17	72.6	346.5	35	-1.1	8.2	4	20.9	0	100
	SP	10	73.7	346.6	35	0.1	9.9	4.7	21.3	0	100
NA20	FL	3	70	346.7	35	1.7	7	3.3	20	100	100
	LN	14	73.4	354.4	35	2.8	7.8	3.8	20.2	14.3	100

### Brain and immune tissue envelopes sometimes differ in length and potential N-linked glycosylation sites

**Figure 4:**
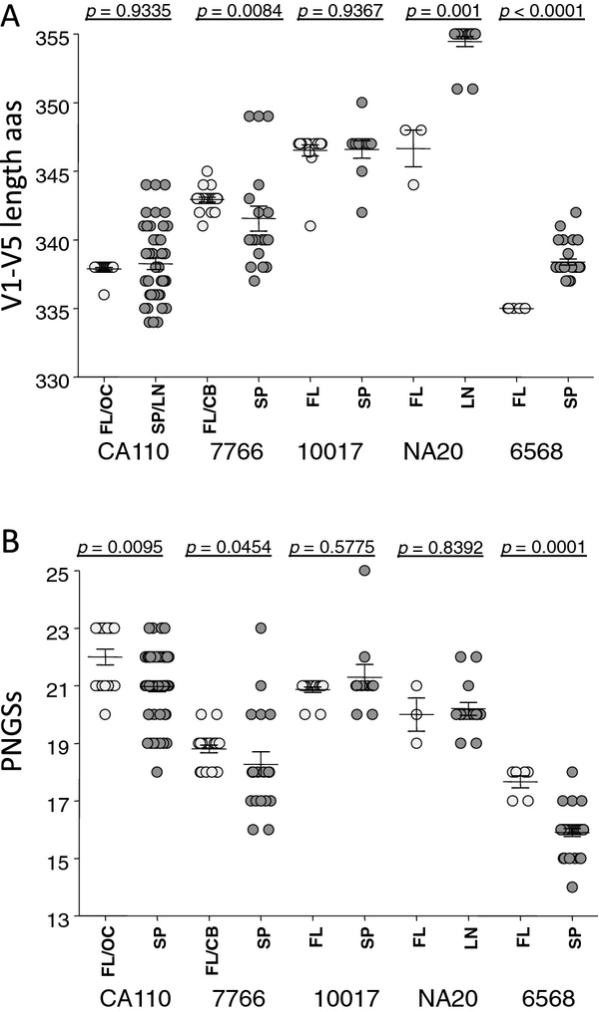
**Brain and immune tissue V1-V5 gp120 do not significantly differ in length (A) and or extent of N-linked glycosylation sites (B)**. All amplified envelopes shown in Figure 1 were included in this analysis.

There were significant differences in V1-V5 length between brain and immune tissue envelopes for subjects 7766, 6568 and NA20 but not for other subjects (Figure 4A, Table 2). However, differences in V1-V5 lengths for subjects 6568 and 7766 brain and spleen envelopes were reversed, while only three NA20 brain envelopes were available for testing. Similarly, no clear pattern between V1-V2 length and brain, immune tissue or macrophage infectivity emerged (Table 2).

There were some differences in the number of potential N-linked glycosylation sites (PNGSs) between brain and immune tissue envelopes (Figure 4B, Table 2). For subjects CA110, 7766 and 6568 those differences were of significance. Curiously, for these subjects, the brain derived envelopes carried a slightly higher average number of PNGSs compared to LN/spleen envelopes.

These observations do not show consistent relationships between either gp120 length or PNGSs sites on gp120 with non-macrophage-tropic or macrophage-tropic phenotypes of R5 envelopes late in disease.

### Envelope signatures for brain and macrophage-tropism

Dunfee *et al. *reported that an asparagine at residue 283 (in the C2 part of the CD4bs; not a glycosylation site) was present in 41% envelopes amplified from brain tissue of subjects with HAD but only in 8% from non-HAD subjects [65]. N283 confers an increased affinity for CD4 [65] and enhances macrophage infectivity [12,65]. Consistent with Dunfee's observations, all the envelopes from NA20 brain tissue contained N283, compared with only 14.3% of those from LN (Table 2). However, all the envelopes amplified from subjects CA110 and 7766 carried N283, while none of those from 6568 and 10017 did. Yet for these four subjects, brain derived envelopes were consistently more macrophage-tropic compared to those from spleen.

Several studies have implicated the glycan at residue 386 in the protection of the CD4bs [66,67] and macrophage infection [67,68]. For subject 6568, all envelopes from spleen carried a PNGS at N386, which was absent in envelopes from brain tissue. For the other four subjects, all envelopes from brain and spleen tissue carried the PNGS at N386.

Together these results show that N283 and the PNGS at N386 sometimes associate with macrophage-tropism and a brain origin. However, this is clearly not a universal relationship.

### Sensitivity of envelopes to the CD4bs mab, b12, soluble CD4 and maraviroc

**Figure 5:**
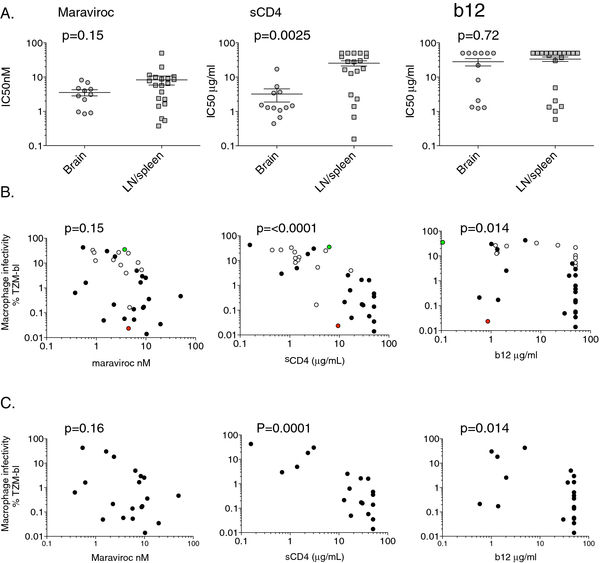
**Sensitivity of brain and immune tissue envelopes to inhibition by soluble CD4, the CD4bs mab, b12 and the CCR5 antagonist maraviroc: The impact of macrophage tropism**. A panel of 32 envelopes that represented the full range of macrophage-tropism observed for LN/spleen envelopes and included highly macrophage-tropic envelopes from the brain was tested for their sensitivity to sCD4, b12 and maraviroc using env+ pseudovirions. A. Brain envelopes were significantly more sensitive to sCD4 compared to those from immune tissues, but are not significantly more sensitive to maraviroc or the CD4bs mab, b12. B. Soluble CD4 sensitivity correlates with macrophage infectivity and is highly significant. There is a weaker correlation between b12 sensitivity and macrophage infection. Control envelopes JR-FL (mac-tropic) and JR-CSF (non-mac-tropic) are depicted as green and red spots respectively. C. Even when just LN/spleen envelopes are analyzed, there is a strong correlation between macrophage infection and sCD4 sensitivity as well as a weaker association with b12. This latter observation rules out the possibility that brain or LN/spleen founder effects affect the correlations shown.

We previously reported a significant correlation between macrophage tropism and sensitivity to sCD4 inhibition for a large panel of envelopes derived from brain and immune tissues as well as from blood and semen [52]. However, there was no significant correlation between macrophage infection and sensitivity to CCR5 antagonists, TAK779 and SCH350581, even though for two subjects, envelopes from brain were significantly more sensitive than those from lymph node [52]. In this previous study, we also observed a trend between macrophage-tropism and sensitivity to the CD4bs mab, b12, although it did not reach significance. These observations support an enhanced interaction between the envelope and CD4 (but not CCR5) for highly macrophage-tropic R5 variants. In contrast to our data, Dunfee *et al. *reported a significant association between enhanced macrophage-tropism in the brain and b12 sensitivity but did not find a relationship with sCD4 sensitivity [69]. To further investigate envelope interactions with CD4 and CCR5, we selected a subset of 32 envelopes from brain and immune tissue of the five subjects (Table 3) along with control non-macrophage-tropic JR-CSF and macrophage-tropic JR-FL envelopes and tested their sensitivity to b12, sCD4 and maraviroc (Figure 5). This set of envelopes was carefully selected to include representatives from each subject that covered the full range of macrophage infectivity observed in LN/spleen. Brain envelopes included were all highly macrophage-tropic except for FL19-49-26, which did not infect macrophages.

**Table 3:** Brain and immune tissue envelopes selected for inhibitor analyses

Subject	Tissue	Envelop	Macrophage infectivity
6568	Frontal lobe	FL 11-1-249	+++
		FL5-47-202	+++
			
	Spleen	SP 12-23-41	+++
		SP 12-24-49	-
		SP 6-22-81	-
		SP 12-14-25	-
		SP 6-25-105	-
			
10017	Frontal lobe	FL24-11-83	+++
		FL27-1-1	+++
			
	Spleen	SP10-9-65	
		SP22-15-107	
		SP9-8-57	
		SP20-12-82	
			
7766	Frontal lobe	FL 19-49-26	+++
		FL 19-54-50	+++
		FL 19-56-66	-
			
	Spleen	SP 13-12-145	-
		SP 13-33-41	-
		SP 15-59--96	+
			
CA100	Frontal lobe	FL 58-13-15	++
		FL 58-30- 92	+++
		FL 56-33-33	+++
			
	Spleen	SP 53-6-122	-
		SP 52-20-75	-
		SP 52-16-50	-
		SP 14-33-34	++
			
NA20	Frontal lobe	B59	+++
		B501-26	+++
			
	Spleen	LN8	-
		LN10	-
		LN14	-
		LN23-14-2	+++
			
			
JR	Frontal lobe	JRFL	+++
			
	Spleen	JRCSF	-

Our new data confirm that brain-derived envelopes were significantly more sensitive to sCD4 inhibition compared to those from LN/spleen (Figure 5A). However, we did not detect significant differences between brain and LN/spleen envelopes for maraviroc or b12 sensitivity (Figure 5A).

We next compared maraviroc, sCD4 and b12 sensitivity to macrophage infectivity (Figure 5B). As we previously reported [52], there was a highly significant correlation between macrophage infectivity and sCD4 sensitivity, but not with maraviroc. A weak correlation with b12 was detected, whereas previously only a trend was noted [52]. In addition, all envelopes tested (except for the CXCR4-using 22-15-107) were completely inhibited by maraviroc (data not shown), i.e. there was no reduction in the level of maximal inhibition that has been associated with use of drug occupied CCR5 [70] or the use of an additional alternative coreceptor.

It needs to be emphasized that the compartmentalization of brain and immune tissue envelopes can lead to the segregation of different phenotypes that reflect the properties of the founding viruses, rather than a specific adaptation to the tissue. To avoid this possibility, we also evaluated whether the variation in macrophage infectivity observed solely among LN/spleen envelopes correlated with sensitivity to sCD4, b12 or maraviroc. The *p *values obtained for each inhibitor were similar to those obtained using the full panel of brain and immune tissue envelopes. Our results therefore confirm a strong correlation between macrophage infectivity and sCD4 sensitivity and a less significant relationship with b12 (Figure 5C).

## Discussion

Our data show for the first time that macrophage-tropism and increased envelope charge among HIV-1 R5 envelopes evolve independently in brain and immune tissue. We also confirm and greatly extend our previous studies that had indicated the compartmentalization of envelope phenotype with macrophage-tropic envelopes in brain tissue and a predominance of non-macrophage-tropic envelopes in immune tissue and plasma [12,13]. The predominance of charged envelopes in immune tissue is consistent with an adaptation for replication in CD4+ T-cells and perhaps colonization of T-cells expressing lower levels of CCR5 late in disease [64]. We are currently investigating these possibilities.

Here, envelope sequences from brain and spleen (or lymph node) derived envelopes were compartmentalized for 4 of 5 subjects. Subject 10017 was clearly an exception, where several of the spleen envelopes clustered with those from frontal lobe. Nevertheless, for all subjects (including 10017), macrophage-tropism was consistently highly compartmentalized to brain tissue. In contrast, the vast majority of envelopes from spleen or lymph node were substantially less macrophage-tropic, varying from background levels of infectivity to moderate infection with strong macrophage infection conferred by only a minority of envelopes. The consistent compartmentalization of envelope phenotype (even in the absence of sequence segregation) is consistent with the presence of strong environmental pressures in the brain and immune tissues selecting for highly macrophage-tropic and non-macrophage-tropic envelopes respectively.

A small number of highly macrophage-tropic envelopes were detected in immune tissue. However, we do not have categorical evidence that they evolved there. The highly macrophage-tropic envelopes from the spleen of subject 10017 clustered among brain-derived envelopes and have not originated independently. The highly macrophage-tropic envelope 23-14-2 from subject NA20 LN clustered with those in lymph node. Our initial interpretation was that macrophage-tropism of this envelope may have evolved independently from those in brain. However, subsequent analyses via Simplot revealed that this envelope was a recombinant and carried sequences between the V1 and V3 loops more closely related to those in brain derived envelopes (data not shown). This observation adds further support to Brown's recent report on intercompartment recombination modulating tropism and contributing to diversity and with affects on tropism [71]. For subjects 7766, 6568 and CA110, a clear gradation of macrophage-tropism was observed in immune tissue with the most macrophage-tropic envs conferring moderate levels of infectivity (although less than the highly mac-tropic brain envelopes). The range of macrophage infectivity in immune tissue of these subjects represents clearer evidence of an independent route to macrophage-tropism compared to brain envelopes. Only very few non-macrophage-tropic envelopes were amplified from the brain tissue samples. These included 51-4-4 from subject CA110 frontal lobe and 31-2-9 from 10017 frontal lobe. These envelopes may have been amplified from T-cells circulating in brain blood capillaries.

Envelope determinants previously reported to be associated with macrophage-tropism include (1) N283 in the CD4bs, which confers an increased affinity for CD4 [65] and enhances macrophage infectivity [12,65]. (2) The loss of an N-linked glycosylation site at residue 386 (N386) [67,68] and (3) a conserved V1 loop residue [72]. However, none of these determinants fully explain the macrophage-tropism of the envelopes studied here. Only for subject NA20, did N283 segregate predominantly with brain envelopes. Curiously, while all envelopes amplified from subjects CA110 and 7766 carried N283, none of those from 6568 and 10017 did. Yet for these four subjects, brain derived envelopes were consistent in conferring substantially higher levels of macrophage infection compared to those from spleen regardless of the presence or absence of N283. All eight brain-derived envelopes from subject 6568 lacked N386 consistent with the reported association. However, all other envelopes amplified in this study contained N386, indicating that its loss is subject dependent. A recent study describing highly macrophage-tropic envelopes amplified from the CSF, also failed to identify determinants of tropism [73]. Further investigation is needed to provide a clearer view of the envelope changes in structure and function that contribute to macrophage-tropism.

Finally, studies from other groups have implicated a more efficient or altered engagement with CCR5 for macrophage-tropic R5 envelopes including those in the brain [51,74]. However, this has not been evident in our studies using maraviroc here or other CCR5 antagonists in previous studies [52]. Nor have we detected consistent differences in infectivity assays using HeLa cell lines expressing different levels of CCR5 [12,13]. Nevertheless, further experiments are needed to evaluate whether macrophage-tropic R5 envelopes (described here) exploit distinct regions of CCR5.

HIV-1 R5 viruses that are non-macrophage-tropic are preferentially transmitted [1-4,75,76]. Data presented here indicate that they still predominate in immune tissue even in late disease. However, increased T-cell tropism (either via CXCR4-use or via retention of CCR5-use [14-17]) and enhanced macrophage-tropism [9-11] are likely to be independent indicators perhaps associated with distinct pathogenic outcomes.

## Conclusions

We show that highly macrophage-tropic, low positive charge envelopes can be detected in brain tissue of AIDS patients suffering from severe neurological disease. These highly macrophage-tropic envelopes contrast sharply with those present in immune tissue that mainly confer inefficient macrophage infection. These latter envelopes carry a higher positive charge that was previously associated with increased T-cell tropism. Strikingly, these distinct phenotypes were evident for subject 10017, even though sequences for this subject were closely related and not clearly compartmentalized. Our data are consistent with the presence of powerful selective environments in immune and brain tissue that select for R5 virus variants with very different characteristics and tropism properties.

## Methods

### HIV-1+ subjects

Brain frontal and occipital lobes, cerebellum, together with spleen and LN samples were obtained at autopsy and kept frozen at -80°C. Tissue samples were provided by the National NeuroAIDS Tissue Consortium (NNTC) and by the University of Edinburgh Brain Bank. HIV-1 envelopes described here were amplified from subjects 7766, 6568, CA110, 10017 and NA20; heterosexual patients with HIV associated dementia (HAD), HIV encephalitis (HIVE) or cognitive impairment all of whom progressed to AIDS and died. Table 1 lists the five patients included for this study. Samples from three subjects provided by the NNTC are from patients treated extensively by HAART (7766, 6568 and 10017), while the fourth (CA110) had no reported use. NA20 was described previously and was a subject from before the HAART era [12,13]. We have increased the number of functional envelopes amplified from this subject.

### Nucleic acid extraction and PCR amplification of env from single molecule templates

Total DNA was purified from tissues using the QIAamp DNA Mini kit (Qiagen), according to the manufacturer's instructions. DNA was eluted in nuclease-free water and frozen immediately at -20°C for storage until analysis.

Sequences covering *rev *and *env *genes were amplified by nested PCR from proviral and circular (episomal) forms of viral DNA present in brain and spleen or LN tissue. The presence of rev in cis upstream from envelope is important for efficient envelope expression and production of high titer env+ pseudovirions. PCRs were carried out using serially diluted tissue DNA samples so that envelopes subsequently cloned were derived from single genomes when 30% or less of PCR reactions were positive. PCRs used high fidelity DNA polymerases (Platinum^®^Taq DNA Polymerase; Invitrogen Inc., or Phusion™ DNA Polymerase; Finnzymes Inc.). PCRs were set up as described previously [12] using the following primers. For proviral amplification, outer primers were RevenvA (5'-TAGAGCCCTGGAAGCATCCAGGAAG-3') and EnvN (5'-CTGCCAATCAGGGAAGTAGCCTTGTGT-3'). Outer primers for episomal DNA amplification were RevenvA and LAI (5'-GCGCTTCAGCAAGCCGAGTCCT-3') [56]. The inner primers were the same for both proviral and episomal rev-env amplification as follows; RevenvBTOPO (5'-CACCTAGGCATCTCCTATGGCAGGAAGAAG-3') and Env-lo (5'-GTTTCTTCCAGTCCCCCCTTTTCTTTTAAAAAG-3'; [77]). The PCR products in positive wells at endpoint dilutions were purified from a 0.8% crystal violet stained agarose gel using a QIAquick Gel Extraction Kit (Qiagen).

All envelopes from subjects CA110, 7766, 6568 and 10017 were amplified and cloned using limiting dilution protocols [2,78]. NA20 envelopes, (including previously described B59, B76, B501, LN8, LN10, LN14 and LN16 [12,13] and newly amplified envelopes, (23-14-2, 23-14-3, 23-14-4, 23-14-9, 23-15-18, 23-15-23, 23-15-28, 23-16-38, 23-17-52 and 23-17-54, 23-15-28) were cloned from proviral DNA not rigorously diluted to endpoint due to limited amounts of DNA.

### Envelope cloning and sequencing

Purified PCR products were cloned into pcDNA™ 3.1D/V5-His-TOPO^® ^(pcDNATM3.1 Directional TOPO^® ^Expression Kit; Invitrogen Inc). Env+ plasmids were transformed into competent *E. coli *(TOP10; Invitrogen Inc.). Colonies were screened for correct rev-env insertions by PCR using a universal T7 Promoter primer (5'-TAATACGACTCACTATAGGG-3') and M5-R (5'-CCAGCTGGGGCACAATAATGTATGGGAATTGG-3' (a primer that hybridizes within our insert); [79]) using Go Taq^® ^Green Master Mix (Promega Inc.). Plasmid DNA was purified using QIAprep Miniprep Kit (Qiagen) and sequenced by Genewiz Inc.

Up to 32 endpoint rev-env clones were sequenced for each sample and analyzed phylogenetically to establish the population diversity. Envelope sequences were analyzed for variation likely to impact on phenotypes e.g. N283, variable loop length, V1-V2, V3 and V1-V5 loop charge, PNGSs as well as for mutations likely to render envelopes non-functional e.g. deletions, premature stop codons, loss of a conserved cysteine involved in disulphide bonding etc. Non-functional envelopes with stop codons or deletions were not included in the analyses.

The nucleotide sequences of novel envelopes reported here have been assigned GenBank accession numbers JN786685-JN786871.

### Phylogenetic analyses

Complete gp160 env nucleotide sequences were assembled and aligned using Clustal × [80] with manual adjustment. All positions with an alignment gap of one or more nucleotides were excluded.

Phylogenetic and molecular evolutionary analyses were conducted using MEGA version 5 [81]. Maximum likelihood phylogenetic trees were generated using General Time Reversible Substitution Model using a discrete Gamma distribution with 5 rate categories and by assuming that a certain fraction of sites are evolutionary invariable for subjects CA110, 7766 and 6568 and without invariant sites for subjects NA20 and 10017. Bootstrap analyses on 1,000 replicates was used to assess the robustness of the tree. Significant (≥ 70%) bootstrap values were assigned to internal tree nodes. Reference sequences representing three HIV-1 group M subtype B http://www.hiv.lanl.gov/ envelopes (FR.83. HXBc2. K03455; TH.90.BK132.AY173951;US.98.1058 11.AY331295) were used as outgroups.

### Cell cultures

293T cells were used to prepare env+ pseudovirions by transfection. HeLa TZM-bl [82] were used to evaluate env+ pseudovirion infectivity titers and neutralization. Pseudovirion infectivity was also evaluated on CD4+ CXCR4+ CCR5- HeLa HIJ cells to monitor CXCR4-use. HeLa TZM-bl cells express high levels of CD4, CCR5 and CXCR4 and contain HIV-inducible β-galactosidase and luciferase reporter genes. 293T cells, TZM-bl cells, and HIJ cells [83], were maintained in Dulbecco's modified Eagle's medium (DMEM, Gibco-Invitrogen, Carlsbad, CA) supplemented with 10% fetal bovine serum (FBS) and were cultured as previously described [12,66,68].

Macrophage cultures were prepared from elutriated monocytes [12,66,68], which were provided by the University of Massachusetts Center for AIDS Research Elutriation Core. The elutriated monocytes were cultured for 5 days in DMEM medium containing 10% human plasma (HP) for differentiation before setting up for infection. Alternatively, macrophages were prepared from Ficoll-purified white blood cells from whole blood by as described previously [12]. On the day prior to infection, the macrophages were washed and resuspended in DMEM medium containing 10% HP and cultured in 48-well tissue culture plates (1.25 × 10^5 ^cells/well/0.5 ml).

### Production and infectivity assays of *env*^+ ^pseudovirions

Env+ pseudovirions were prepared by cotransfection of env+ pTOPOenv vector with env- pNL4.3Δenv construct that carried a premature stop codon in *env *[12] into 293T cells using calcium phosphate. Cell free supernatants were harvested after 48 h culture and frozen at -152°C prior to experimental analysis.

Env+ pseudovirions were titrated on HeLa TZM-bl cells, HeLa HIJ cells and on macrophages. For HeLa TZM-bl and HeLa HIJ cells, 2 × 10^4 ^cells/0.5 ml were added to each well on a 48 well plate the day prior to virus titration. Virus titers were determined as described previously (Peters 2004, 2006). Briefly, 100 μl of serially diluted viral supernatants in DMEM media (10% FBS) were added to cells in duplicate and incubated for 3 hours. 0.4 ml of DMEM (10% FBS) was added to each well and cultures incubated for 48 h (Hela TZM-bl) or 72 h (HIJ). TZM-bl cells were then fixed in 0.5% gluteraldehyde in PBS and β-galactosidase X-gal substrate added. HeLa HIJ cells were fixed in cold methanol:acetone 1:1, washed and immunostained for p24 using monoclonal antibodies 38:96K and EF7 (UK Centre for AIDS Research), followed by an anti-mouse IgG-β-galactosidase conjugate and X-gal substrate (0.5 mg/ml X-gal, 3 mM potassium ferrcyanide, 3 mM potassium ferrocyanide, 1 mM magnesium chloride).

Macrophages were seeded in 48 well plates (1.25 × 10^5 ^cells/0.5 ml/well) the day prior to infection. Macrophages were pretreated with 0.1 ml DEAE dextran (10 μg/ml) in DMEM medium containing 10% HP for 30 min at 37°C before virus supernatants were added and spinoculating for 45 minutes in a benchtop centrifuge [84]. Infected macrophages were incubated for a further 3 h at 37°C before the addition of 0.4 ml of DMEM (10% HP) and incubating at 37°C for seven days. Macrophages were then fixed and immunostained for p24 as described for HIJ cells. DEAE dextran and spinoculation enhance virus infectivity by approximately 20-fold by increasing attachment [84] and entry [85]. Infection following this procedure does not bypass the requirement of CD4 and CCR5 for infection, which remains sensitive to entry inhibitors. Thus, macrophage infection conferred by envelopes described here was inhibited by maraviroc (not shown). Since env^+ ^pseudovirions are capable of only a single round of replication, we were able to estimate the number of focus-forming units (FFU) by counting individual or small groups of infected blue-stained cells by light microscopy. Average numbers of FFUs/ml were then calculated. All values represent the averages of at least two independent experiments, each done in duplicate and using macrophages from different donors. Error bars in figures were calculated from replicate wells of both experiments.

Each set of macrophage, TZM-bl and HIJ infections included several control env+ pseudovirions including NL4.3 (X4), JR-CSF and NA420 LN40 (non-mac-tropic R5 envelopes), JR-FL and NA420 B33 (mac-tropic R5 envelopes).

### Inhibition and neutralization assays

Inhibition and neutralization assays for soluble CD4 (sCD4), maraviroc and mab b12 were carried out in 96 well plates as described previously using HeLa TZM-bl cells as target cells [52] For maraviroc, cells were treated with 2-fold dilutions in 50 μl for 30 minutes before adding an equal volume containing 200 FFU of pseudovirions. For sCD4 and b12, 50 μl samples of serially diluted sCD4 were mixed with 50 μl env+ pseudovirions carrying 200 FFU at 37°C for 1 h and added to HeLa TZM-bl cells. To evaluate residual infectivity, medium was removed and 100 μl of medium without phenol red added. Cells were then fixed and solubilized by adding 100 μl of Beta-Glo (Promega Inc.). Luminescence was then read in a BioTek Clarity luminometer.

### Statistical methods

A robust semiparametric regression model [86] implemented by *R *package *drc *[87] was used to model dose response relationships between the macrophage infectivity (as a percentage of TZM-bl infectivity) and IC50 concentration of maraviroc, sCD4 and b12. The dosages that caused 50% inhibition (IC50) were estimated as well as their 95% confidence intervals (not shown). Two parameter log-logistic regressions were used for one envelope, FL11-1-249 for b12 inhibition due to over-fitting of semiparametric approach in the neighbor area of IC50. When there was no apparent inhibition or inhibition failed to reach 100%, the model-fitting algorithm either did not converge or reported an extrapolating estimate out of the experimental ranges with a very wide confidence interval. For these inhibitions, the IC50 estimates were winsorized by defining them manually from Excel plotted graphs. Two tailed, nonparametric Mann Whitney tests were used to evaluate whether there exists statistically significant differences between distributions of IC50s of maraviroc, sCD4 and b12 for envelopes from brain and from LN/spleen. Two tailed, non-parametric Spearman tests were used to evaluate whether there exists monotonic correlation between macrophage infectivity and IC50s for maraviroc, sCD4 and b12 (Figure 5). Two tailed, nonparametric Mann Whitney tests were also used to test for significant differences in V1-V5 positive charge, length and number of potential N-linked PNGSs sites. These tests were carried out using Graphpad Prism 5 for Mac OSX.

## Competing interests

The authors declare that they have no competing interests.

## Authors' contributions

MPG-P amplified the envelopes, carried out the phylogenetic studies and wrote the manuscript with PRC. OO'C did the neutralization and inhibition assays. RL analyzed the neutralization and inhibition data and derived IC50s. WMS amplified some of the initial envelopes in this study. JB provided patient information and advice on samples. PS provided advice on phylogenetic analyses. PRC conceived the study and wrote the manuscript with MPG-P. All authors provided comments on the manuscript. All authors read and approved the final manuscript.
